# Incidental Paratubal Non‐Functional Adrenal Rest in an Adult Female: A Rare Histopathological Finding

**DOI:** 10.1002/ccr3.72621

**Published:** 2026-04-24

**Authors:** Amir Hosein Jafarian, Parisa Vedad, Zahra Zohani, Sarvin Beigi

**Affiliations:** ^1^ Department of Pathology Mashhad University of Medical Sciences Mashhad Iran

**Keywords:** adrenal rest, case report, ectopic adrenal tissue, histopathology, incidental finding, paratubal adrenal tissue

## Abstract

Paratubal adrenal rests, although rare in adult females, should be considered during histopathological evaluation of adnexal tissues, as they may mimic neoplasms or rarely undergo functional or pathological change. Surgeons should be aware of this possibility to avoid unnecessary concern or overtreatment when encountering small yellow nodules intraoperatively.

## 1 Introduction

Adrenal rests are ectopic adrenal tissues in various locations [1]. The adrenal glands have a dual embryonic origin: the cortex is derived from the mesodermal epithelium, whereas the medulla originates from the neuroectoderm [2]. Adrenal rests may consist solely of cortical tissue or a combination of both cortical and medullary tissue, depending on whether the fragments detach before or after the migration of neural medullary tissue into the cortex [1]. This ectopic tissue can occur in various locations, including the retroperitoneum and the genitourinary tract and can be found in up to 50% of infants but typically undergoes atrophy and disappears within a few years, persisting in less than 1% of the adult population. They are more common in men and very rare in women [3].

Most adrenal rests are clinically silent and discovered incidentally. However, they may undergo hyperplasia, neoplastic transformation, or become functionally active, leading to endocrine manifestations or mass effects [3, 4, 5]. Given the rarity of paratubal adrenal rests in adult women and their capacity to mimic neoplastic lesions, reporting such cases is essential to enhance diagnostic recognition and prevent potential misinterpretation.

## 2 Case History/Examination

A 47‐year‐old Iranian woman presented with several months of heavy menstrual bleeding and abdominal discomfort. Pelvic ultrasound revealed multiple leiomyomas within the uterine wall. The patient subsequently underwent total hysterectomy and bilateral salpingectomy. No preoperative endocrine evaluation was performed as the patient had no clinical features suggestive of hormonal excess.

**FIGURE 1:**
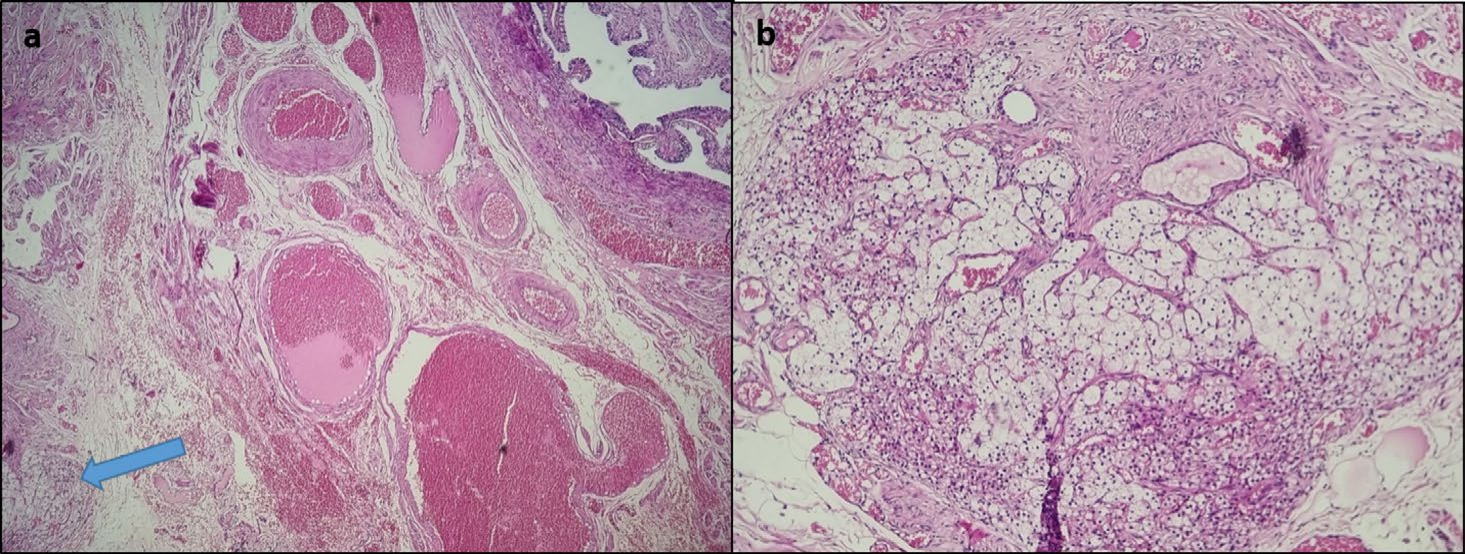
Microscopic examination of the fallopian tube. Pratubal region, Arrow points to the Adrenal rest (a) (H&E stain, original magnification: ×40), Adrenal rest (b) (H&E stain, original magnification: ×100).

Gross examination revealed multiple intramural and subserosal leiomyomas. Histopathological evaluation confirmed conventional leiomyomas. Notably, in the paratubal region, an incidental finding of an unencapsulated adrenocortical nodule composed of zona glomerulosa and zona fasciculata without atypia and mitosis was observed (Figure 1).

## 3 Differential Diagnosis, Investigation and Treatment

The main differential diagnosis included ectopic adrenal tissue, ovarian steroid cell tumor, and metastatic lesion [6]. Preoperative imaging (ultrasound) had not detected the lesion due to its small size (approximately 3 mm). Histopathological examination revealed two cell populations resembling zona glomerulosa (eosinophilic cytoplasm, centrally placed hyperchromatic nuclei) and zona fasciculata (larger cells with abundant clear cytoplasm) of adrenal cortex. No atypia, mitotic activity, or necrosis was identified. The findings were consistent with adrenal rest tissue. Immunohistochemistry was not required due to the characteristic histomorphological features and absence of atypia.

No further treatment was required, as the lesion was completely excised and no evidence of adrenal hormone excess or deficiency was observed during follow‐up visits.

## 4 Conclusion and Result

The patient was discharged two days after surgery and remained asymptomatic at follow‐up, with no clinical signs of adrenal hormone excess or deficiency, supporting the diagnosis of a non‐functioning lesion.

Paratubal adrenal rests are rare in adult women. They are usually asymptomatic and seldom demonstrate biological or pathological activity. We report this case to highlight the incidental finding of a rare phenomenon that should be considered in the differential diagnosis of paratubal masses.

Recognition of paratubal adrenal rests is essential to avoid misdiagnosis and unnecessary intervention, particularly in adult female patients undergoing surgery for unrelated gynecologic conditions.

## 5 Discussion

Paratubal adrenal rests represent a rare form of ectopic adrenal tissue located adjacent to the fallopian tube [5]. Morgagni first described adrenal rests in 1740 as yellowish nodules adjacent to the normal adrenal gland. They are considered remnants of adrenal cells that failed to regress during embryogenesis. Recent studies suggest that ectopic adrenal tissue is present in up to 50% of newborns, but it typically regresses during early childhood. Consequently, its prevalence in adults is estimated to be below 1% [7].

These lesions are usually discovered incidentally during pelvic and inguinal surgeries, as most patients remain asymptomatic. In two case studies, ectopic adrenal tissue in the inguinal canal was found incidentally in six and four young boys over two decades and 18 months, respectively. Most cases were associated with undescended testes, and all were benign with no clinical consequences [3, 8, 9].

Although paratubal adrenal rests are benign, their clinical significance arises in two main contexts:

1. They may be hormonally active, leading to clinical symptoms or endocrine disorders, and are capable of developing the same pathologies as the adrenal gland [3].
2. They may mimic neoplastic lesions, posing a diagnostic challenge [3, 4, 6, 10].

Diagnosis is usually based on architectural and cytological features, supplemented by immunohistochemistry when necessary [11].

This case reinforces the importance of careful histological evaluation of adnexal tissues, as incidental adrenal rests may be overlooked or misinterpreted as neoplastic lesions.

## Author Contributions

**Amir Hosein Jafarian:** data curation, investigation, project administration. **Parisa Vedad:** data curation, formal analysis, methodology, project administration, software, supervision, validation, writing – original draft, writing – review and editing. **Zahra Zohani:** methodology, supervision, validation. **Sarvin Beigi:** data curation, investigation, project administration, software, writing – original draft, writing – review and editing.

## Funding

The authors have nothing to report.

## Ethics Statement

The authors have nothing to report.

## Consent

The authors declare that written informed consent was obtained for the publication of this manuscript and accompanying images using the consent form provided by the journal.

## Conflicts of Interest

The authors declare no conflicts of interest.

## Data Availability

Data and material are available on reasonable request from the corresponding author.
